# Effects of Exercise on Cognitive Performance in Children and Adolescents with ADHD: Potential Mechanisms and Evidence-based Recommendations

**DOI:** 10.3390/jcm8060841

**Published:** 2019-06-12

**Authors:** Lasse Christiansen, Mikkel M. Beck, Niels Bilenberg, Jacob Wienecke, Arne Astrup, Jesper Lundbye-Jensen

**Affiliations:** 1 Department of Nutrition, Exercise & Sports, University of Copenhagen, 2200 Copenhagen, Denmark; mib@nexs.ku.dk (M.M.B.); wienecke@nexs.ku.dk (J.W.); ast@nexs.ku.dk (A.A.); jlundbye@nexs.ku.dk (J.L.-J.); 2 Research Center in Neurodevelopmental Disorder (FOCUS), Child and Adolescent Mental Health Odense, Mental Health Services Region of Southern Denmark, 5000 Odense, Denmark; Niels.Bilenberg@rsyd.dk; 3 Institute of Clinical Research, University of Southern Denmark, 5000 Odense, Denmark

**Keywords:** ADHD, exercise, cognition, executive functions, physical activity

## Abstract

Attention Deficit Hyperactivity Disorder (ADHD) is a neurodevelopmental disorder with a complex symptomatology, and core symptoms as well as functional impairment often persist into adulthood. Recent investigations estimate the worldwide prevalence of ADHD in children and adolescents to be ~7%, which is a substantial increase compared to a decade ago. Conventional treatment most often includes pharmacotherapy with central nervous stimulants, but the number of non-responders and adverse effects call for treatment alternatives. Exercise has been suggested as a safe and low-cost adjunctive therapy for ADHD and is reported to be accompanied by positive effects on several aspects of cognitive functions in the general child population. Here we review existing evidence that exercise affects cognitive functions in children with and without ADHD and present likely neurophysiological mechanisms of action. We find well-described associations between physical activity and ADHD, as well as causal evidence in the form of small to moderate beneficial effects following acute aerobic exercise on executive functions in children with ADHD. Despite large heterogeneity, meta-analyses find small positive effects of exercise in population-based control (PBC) children, and our extracted effect sizes from long-term interventions suggest consistent positive effects in children and adolescents with ADHD. Paucity of studies probing the effect of different exercise parameters impedes finite conclusions in this regard. Large-scale clinical trials with appropriately timed exercise are needed. In summary, the existing preliminary evidence suggests that exercise can improve cognitive performance intimately linked to ADHD presentations in children with and without an ADHD diagnosis. Based on the findings from both PBC and ADHD children, we cautiously provide recommendations for parameters of exercise.

**Preface**

ADHD is a neurodevelopmental disorder characterized by inattention and/or hyperactivity and impulsivity diagnosed in children before the age of 12 [1]. Worldwide prevalence of ADHD in children and adolescents is estimated to be between 5% and 10% [2,3,4] and recent surveys estimate that 57% of diagnosed cases persist into adulthood [5], with well-documented detrimental impact on social and academic skills [6]. The gravity of this diagnosis is underlined by the decreased life expectancy [7], comorbidity with other psychiatric diagnoses [8] and substance abuse [9], along with reduced quality of life for the affected children and their families [10]. Present treatment approaches most often include stimulant and non-stimulant pharmacotherapy along with cognitive therapy. Stimulants such as methylphenidate (MPH) show the largest positive effects on the symptomatology in ADHD, but 20–25% of the diagnosed individuals do not respond to the treatment, and pharmacotherapy can be accompanied by physical and psychological adverse effects [11,12,13,14,15,16,17,18]. The gravity and prevalence of ADHD thus urgently calls for therapeutic approaches which can supplement or potentiate the effect of pharmacological and cognitive treatment as well as improve the life for children resilient to these. Physical activity may constitute such an approach. 

For a child to be diagnosed with ADHD, she or he must present at least six of the nine symptoms within either the inattentive and/or the hyperactivity and impulsivity category. According to the American Psychiatric Association’s Diagnostic and Statistical Manual for Mental disorders (DSM-5) [1], a child can be diagnosed with either of the three ADHD presentations: inattentive, hyperactive and impulsive or some combination of these [1]. ADHD is thus an umbrella diagnosis. Whereas categorical diagnoses are of clinical and practical value, they may conversely oversimplify a complex mixture of cognitive traits. This appears to be the case for ADHD, where the current evidence demonstrates that ADHD should be considered as the impairing tail of traits that vary continuously throughout the general population rather than a discrete category [19,20,21,22,23]. This suggests that interventions or activities, which benefit children, in general, are also relevant for children and adolescents with ADHD and may alleviate the ADHD symptomatology. Acute and regular physical activity (PA) are known to trigger a wide array of physiological events that can lead to improvements in physical and psychological well-being as well as physical and cognitive functions including the memory domain (see e.g., [24] or [25] for review). In contrast, the effects of PA on cognitive functions, which are known to be affected in children with ADHD compared to population-based controls (PBC) are sparsely studied. In this review, we describe deficits in cognitive performance associated with ADHD and review the empirical evidence suggesting effects of exercise on cognitive performance (specifically Executive Functions (EFs) including selective attention as well as sustained attention) in children with and without ADHD. To substantiate the discussion, we characterize neurophysiological and neurocognitive abnormalities associated with ADHD and provide a mechanistic framework for the counteracting or ameliorating effects of exercise in children and adolescents while highlighting key findings from the recent decades of research. Furthermore, we extract and present effect sizes from existing intervention studies and discuss these results in light of the neurophysiological framework presented. Finally, we will cautiously provide recommendation of duration, intensity, and type of acute and long-term exercise interventions for persons diagnosed with ADHD. 

## 1. ADHD, Cognitive Functions and the Ameliorating Effects of Exercise

The cardinal symptoms of inattentive ADHD encompass distractibility, forgetfulness, poor organization skills and low perseverance, whereas hyperactivity and impulsivity are associated with impatience for delayed rewards, difficulties in inhibition of untimely and inappropriate motor responses along with inability to dampen motor activities to appropriate levels for a given situation [1]. The link between these symptoms and executive functions is unmistakable [26], and accordingly, existing research has documented performance deficits in tests of cognitive (incl. executive) functions in children with ADHD [27,28,29]. Three primary aspects of executive functions (EF) are traditionally identified; working memory, inhibitory control and cognitive flexibility or set shifting [26,30]. Children with ADHD display subpar performance in some, but not all aspects, and rarely the same tests of EFs (see e.g., [31] for review). Every child thus has his or her own profile, i.e., a combination of performance in tests of EFs and attentional control, which reflects his or her idiosyncratic etiology. Importantly, this individual combination of performances across several tests of NCFs may entail pivotal information about his or her individual path to remission. The framework for this review is thus that of assessing the effects of exercise on performance in tests of EFs and attention in children and adolescents with (and without) ADHD. The objective is to answer the question: To what extent can exercise improve cognitive deficits associated with pediatric ADHD and which modality, intensity and duration of exercise might benefit cognitive performance based on studies of acute and long-term exercise in children and adolescents with and without ADHD? 

### 1.1. ADHD is Associated with Lower Performance in Cognitive Tests

Although ADHD is in general associated with lower performance, neuropsychological tests are in isolation unlikely to have sufficient selectivity necessary to entail clinical value [32]. As an example, if pediatric ADHD was diagnosed based on impaired performance (>90 percentile) in at least five commonly used tests for executive functions, ~10% would be identified, whereas ~50% would be identified using only the most sensitive of these (see [33] for a discussion). Additionally, several of the tests commonly deployed to assess EF in ADHD have low construct validity (see e.g., [34] for a discussion). At first sight, the tenet that deficits in EF may lead to the complex array of ADHD symptoms appears to be scarcely supported in the literature. As an example, meta-evidence suggests that impaired inhibitory control [35], which is thought to be a fundamental deficit in ADHD, does not differ between individuals with ADHD and age-matched population-based controls when evaluated with the Stroop test [36]. On the other hand, meta-analyses results for both go/no-go [37] and stop-signal [38] tasks reveals impaired inhibition in ADHD. As a result, meta-analyses including all three tests find small differences between PBC and ADHD populations across different tasks and test paradigms [39]. Improving task validity may thus increase sensitivity, and recent results demonstrate that slight protocol alterations can improve validity of tasks probing working memory in ADHD [40]. Furthermore, deploying a comprehensive test-battery including several tests within each EF domain can increase sensitivity and specificity to 89 and 80% respectively [41]. Another important point of criticism is the practice of comparing pooled average performance scores with little attention to intra-individual variability. This may impede detection of subpar performance. Exemplified; The lower selective and sustained attention ability associated with ADHD results in lower average performance (e.g., higher mean reaction times (RT) in a flanker test), which originates in part from large intra-individual variability [42,43,44]. This variability does not stem from large systematic fluctuation in trial-to-trial performance, but rather from few very high RTs, signifying lapses in attention [45]. 

Assessing internal phenomena such as cognitive processes is not straightforward and crude categorization into ‘presentations’ or ‘core symptoms’ may not be of sufficient fidelity to fully describe the idiosyncratic etiology of ADHD. Each core symptom of ADHD assessed through conventional scales (e.g., Connor’s rating scales) is influenced by several neurocognitive functions typically operationalized as performance on neurocognitive tests. Although, each cognitive construct (i.e., ‘working memory’, ‘cognitive flexibility’, ‘inhibitory control’ ‘attentional control’) involves multiple and overlapping neurophysiological processes and testing these independently is accordingly troublesome (e.g., testing WM independent of attentional control), they do provide cut out a middleman and provide a more detailed picture of the pathology. Elucidating the effect of exercise on tests of executive functions and attention (combined termed neurocognitive functions, NCF) provides vital information on how to target exercise interventions for children with ADHD.

Meta-analyses suggest that pediatric ADHD is associated with consistently lower performance in tests of response inhibition, behavioral inhibition, reaction time variability, cognitive flexibility, choice impulsivity (delayed gratification and delay discounting tasks) and WM [29,38,46,47,48]. As argued below, exercise might constitute an avenue to symptom amelioration by improving these NCFs. A raison d’être for this narrative review is therefore that changes in NCFs reflect changes in the clinical severity for children with ADHD. However, correlated improvements in NCFs and ADHD symptomatology suggest that this is not the case for exercise (e.g., [49]). Furthermore, as cognitive functions develop in parallel with CNS maturation during the first two decades of our life [50], it can be argued that e.g., subpar NCFs in pediatric ADHD is solely a matter of tardy cognitive maturation and targeting e.g., EF specifically in children makes little sense [51,52,53]. However, as measures of EF influence learning behaviors more than estimates of core symptomatology [54] and ADHD-related EF deficits are evident in adolescents [55] and persist into adulthood [56], it appears that deficits in NCFs (incl. EF) are of clinical importance and do not wane with central nervous maturation [57]. In the following section, we provide a comprehensive overview of the effect of exercise on tests of NCFs where children with ADHD generally display subpar performance. 

### 1.2. Exercise Benefits Performance in Neurocognitive Tests

Physical activity (PA) and exercise exerts a plethora of beneficial physiological, psychological and neurocognitive effects. These include reductions in stress [58], anxiety [59], depression [60] and negative affect [61] along with positive influences on cognition including improved executive and memory functions (see [24] for review). A discussion of the effects of exercise on cognition warrants a few semantic clarifications. Firstly, physical activity can be defined as any *bodily movement that results in energy expenditure* [62]. This broad definition includes both planned and deliberately executed *exercise* along with everyday activities such as commuting on foot. The distinction between PA and exercise feeds into the perspectives dominating the research field; exercise may act as a neuro-enhancer and improve cognitive performance through acute and long-term effect on monoaminergic transmission, neurotrophic signaling and mechanisms of neuroplasticity (see e.g., [24] for review). Additionally, it is also a prevalent perspective in the literature, that the cognitive load associated with engaging in physical activities and exercise (e.g., decision making processes, adherence to the rules of a game or the demands for flexible behavior as a team member [63]) could entail training of impaired cognitive functions. The possibility of the latter is discussed below. 

#### 1.2.1. Acute Exercise Affects Performance in Tests of Cognitive Functions

During the recent years, there has been an increasing focus on acute effects of a single bout of exercise. Acute exercise increases arousal, which is accompanied by potentiated performance in a wide array of cognitive tasks. Although a single bout of exercise is unlikely to cause long-lasting changes in the cognitive functioning, the immediate effect can be harvested for relevant purposes (e.g., improved class-room behavior and learning outcome). Across cognitive domains, meta-analyses point to positive effect of acute exercise in PBC children. For combined EFs, effect sizes (ESs, either Cohen’s *d* og Hedges’ *g*) vary from 0.19 to 0.54 [64,65,66,67], whereas two meta analyses score effects of exercise on measures of attention to ESs of 0.43 and 0.42 [65,66]. To the best of our knowledge, only four meta-analyses investigating the effect of exercise on cognition including children with ADHD exist [68,69,70,71]. None of these calculate the effect of acute and long-term exercise separately and we accordingly present these at the end of this section. 

A few findings on the effects of acute exercise on PBC children are of particular interest for the present review and warrant further discussion. Dividing PBC children into high and low performance groups based on a preceding Flanker’s Task testing inhibitory control, Drollette et al., found that exercise elicits larger beneficial effects on inhibitory control in the lower performing children [72]. Based on the aforementioned notion that ADHD represents the extremes of traits present throughout the general population, the results suggest that children with ADHD are thus likely to benefit the most from acute exercise. Supporting this, several studies report positive effects of acute exercise on performance on several tests of cognitive functions in children with ADHD. Silva et al. found increased performance in an attention-demanding first person computer simulation [73], but also positive effect on *inhibitory control* has been reported deploying go/no-go [74,75], Flanker [76,77,78] and Stroop [79,80,81] tests. In addition, findings of exercise-induced performance increases for cognitive flexibility probed with modified Flanker tests [77], task-switch tests [82], the Wisconsin card sorting task [81] and the alternate use task [83] have also been reported. Importantly, several contrasting findings also exist [84,85,86,87]. The divergent results likely reflect methodological heterogeneity. For example Craft reported no change in digit span or general intelligence following a bout of exercise lasting 1 to 10 m [85]. It is likely that durations of ~10 m are not sufficient to impact subsequent performance on tests of NCFs. 

In summary, some studies investigating effects of acute exercise have found positive effects on performance in various aspects of cognitive functions – also in children with ADHD. Unless the observed positive effects are merely transient, taken at face value the beneficial effects of acute exercise could in term over the course of weeks or months accumulate into differences in levels of cognitive functioning. Such effects would be reflected in both positive associations between cognition and physical activity levels and changes in cognitive performance after long-term interventions. This is discussed in the subsequent sections.

#### 1.2.2. Association between Fitness or Physical Activity Level and Cognitive Functions

Activity levels of children are typically measured directly as accelerometer data collected over one or more typical days [88], indirectly through physical activity questionnaires [89] or by assessment of the fitness level (e.g., [90,91,92,93,94]). By correlating fitness and activity levels with performance on tests of NCFs, associations between ‘chronic physical activity’ and cognitive functions can be estimated. Evidence from meta-analyses supports a positive relation in PBC children [95,96,97]. Children with ADHD are less likely than their PBC peers to meet recommended levels of physical activity, and based on the aforementioned studies, this may be linked to deficits not only in physical capacity etc., but also in measures of cognitive functions [98]. In a large sample representing the general population (*n* = 45,897), Cook et al. reported that young individuals with ADHD who are unmedicated, are—or are likely to be—sedentary [98]. This supports earlier finding of an increased risk of a sedentary lifestyle and risk of obesity in un-medicated youth with ADHD [99].

In preadolescent children with a high risk of ADHD (i.e., ≥90th percentile on the hyperactive/impulsive parents’ and teachers’ rating scales), Brassell et al., found a positive correlation between aerobic fitness and accuracy for the incongruent trials in a modified Flanker task as well as lower interference scores, altogether signifying better inhibitory capacity for high fit children [100]. In line with this, longer response times across congruent and incongruent trials in the Flanker task has been reported for children with ADHD and low cardiovascular fitness compared to a group of high fitness children with ADHD [101]. Better performance in tests probing executive functions may also relate to activity levels as Gapin et al., found that in a small sample of children with ADHD (*n* = 14) activity levels measured over 7 days with an accelerometer were positively related to *planning* abilities assessed as performance in the Tower of London task [102]. Altogether, the association between PA and cognitive functions across the general population as well as within the pediatric ADHD population appear robust although causal relations cannot be assumed, and reports of contrasting findings exist. 

#### 1.2.3. Long-Term Exercise Improves Performance in Cognitive Tests 

Meta-analyses of the causal effect of long-term exercise interventions cognitive function in PBC children find small effects on EFs (0.24 [65], 0.20 [103,104] and 0.14 [67]), while one analysis reports large effects on attention (g = 0.90) [65]. This effect originates from a single original dataset of 230 children [105] demonstrating improved capacity for processing/psycomotor speed, concentration and attention following 5 months of increased PA assessed by the d2-R test thought to probe sustained attention [106]. 

A positive effect of structured exercise on hyperactive behavior was suggested in 1980 by Allen who found short periods of jogging prior to the beginning of the school day over the course of 6 weeks to improve classroom behavior in twelve boys with unspecified behavioral and gross motor impairments [107]. Within the cognitive domain, several studies have found improvements in one or more measures in children with ADHD following long-term exercise [49,108,109,110,111,112,113,114,115,116,117]. As discussed below, exercise that is both coordinative and cardiovascular challenging may be more engaging (and efficient). As an example, Pan et al. found 24 table tennis practice sessions over the course of 12 weeks to improve behavioral inhibition measured by use of the Stroop test and decreased behavioral problems during the training period [110,111].

In conclusion, long-term studies of the effects of exercise and physical activity on measures on performance in cognitive tests demonstrate positive effects in both PBC children and children with ADHD. It is, however, noteworthy there are only few long-term studies in children with ADHD reporting all exercise parameters. More studies are thus needed in particular in children with ADHD detailing the influence of specific exercise parameterization including timing.

#### 1.2.4. Meta-Analyses Suggest Beneficial Effects of Exercise on Cognitive Functions in Children with ADHD

Four meta-analyses have reported effects of exercise on cognition in (or including) children with ADHD; one combines effects from cross-sectional, acute and long-term investigations [70], one sums evidence from both acute and long-term [71], one include only three studies [69] and one includes participants with autism (although a separate analysis for the ADHD diagnosis was conducted) [68]. Consequently, at present, the methodological heterogeneity between studies hampers the possibility of drawing firm conclusions from meta-analyses. 

In 2015, Cerrillo-Urbina and coworkers presented evidence from eight randomized controlled trials with a total of 249 children [69]. Of the eight studies included in the analysis, seven applied aerobic exercise over a time span ranging from a single session to 10 weeks with significant overall effects on all cognitive domains tested. These inclusion criteria are very broad for both the exercise interventions and cognitive outcome measures, but the overall Effects Size (ES) in support of a positive effect of exercise was (Cohen’s d) 0.84 with ES ranging from 0.56 (hyperactivity and impulsivity) over 0.58 (executive functions), 0.59 (social skills) to 0.66 (anxiety). Supporting positive effects of exercise, Vysniauske et al. reported similar evidence for an alleviating effect of exercise in ADHD. By combining evidence from seven studies they found beneficial effects measures of executive functions (g = 0.54) with a meta-regression pointing to a longer duration of the exercise intervention leading to larger effects [71]. The latter conclusion is however somewhat hampered by the inclusion of both acute and long-term experiments, mixing immediate, transient effects with effects of long-term exercise and these effects should be addressed separately. In addition, several systematic and narrative reviews have provided less objective evaluations of the extant evidence and recommendations for clinical application. Recently, Cornelius et al. summarized and combined evidence from 20 original studies and concluded that physical activity regardless of *intensity, length*, duration and frequency had beneficial effects for children with ADHD (g = 0.81) [70]. Despite moderate to large effect sizes (0.46 to 1.6) neither attention, EFs, academic achievements, social problems or disruptive behaviors differed significantly from control conditions. The overall effect can be accredited to the emotion/mood category. The conclusions should be interpreted with caution due to the combination of evidence from association studies, uncontrolled single-session studies and RCTs in addition to the heterogeneity between studies in extent and type of exercise. 

Notwithstanding the limitations of the available meta-analyses, recent evidence-based practice recommendations suggest moderate to large positive effects of exercise on inattention, impulsivity, hyperactivity and executive functioning [118]. This is supported by conclusions from both systematic and narrative reviews [119,120,121,122,123,124,125]. 

## 2. The Neurophysiology of ADHD and how Exercise may Exert Beneficial Effects

**Figure 1:**
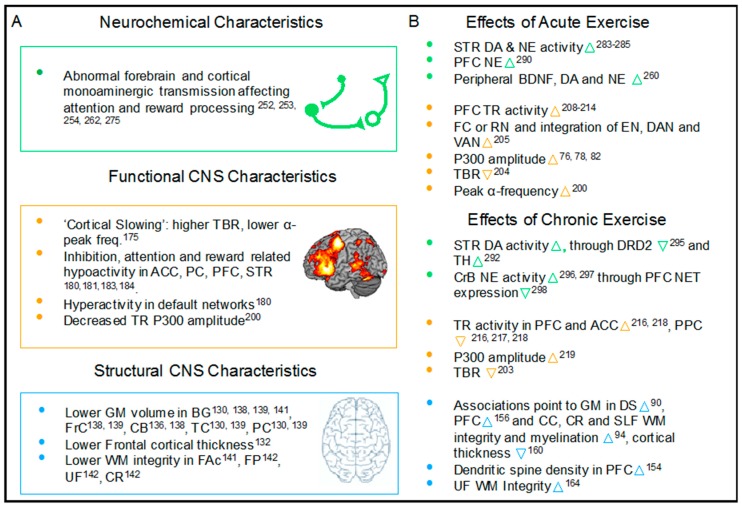
Neurophysiological differences between PBC and children with ADHD and the potential counteractive effects of exercise. (**A**) Identified neurophysiological and anatomical differences between children and adolescent with ADHD and their PBC peers divided into analysis level (‘neurochemical’, ‘functional’ and ‘structural’) in colour-coded boxes. Due to the substantial amount of experimental work conducted on differences in task-related activation between indiviudals with ADHD and PBC peers, the citations all refer to meta-analyses. (**B**) Potential counteracting effects of ‘acute’ and ‘chronic’ exercise are listed in representative colours. Please note that ‘chronic exercise’ encompasses both long-term intervention and associations. Delta and Nabla denote the sign of the physiological change. Abbreviations are listed below. Abbreviations: ACC (anterior cingulate cortex), BDNF (brain-derived neurotrophic factor), BG (basal ganglia), CB (cerebellum), CC (corpus callosum), CR (corona radiata), CrB (cerebral), DA (dopamine), DAN (dorsal attention network), DRD2 (dopamine receptor D2), DS (dorsal striatum), EN (executive networks), FAc (frontoaccumbal) FC (functional connectivity), FP (frontoparietal), FrC (frontal cotex), FT (frontotemporal), GM (grey matter), NE (norepinephrine), NET (norepinephrine transporter), PC (parietal cortex), PFC (prefrontal cortex), PPC (posterior parietal cortex), RN (reward networks), SLF (superior longitudinal fasciculus), STR (striatum), TBR (theta/beta ratio), TC (temporal cortex), TH (tyrosine hydroxylase), TR (task-related), UF (uncinate fasciculus), VAN (ventral attention network), WM (white matter).

As discussed above, ADHD pathology is complex, and several neurophysiological abnormalities contribute to the low performance in test of NCFs. A qualified discussion of how exercise may improve cognitive functions in children with ADHD warrants an outline of differences in central nervous structures, network activity and brain neurochemical signaling between PBC and children with ADHD as well as an overview of how exercise impacts the CNS. See Figure 1 for a schematic illustration summarizing the succeeding sections. 

### 2.1. Brain Structure Abnormalities Associated with Pediatric ADHD and the Effects of Exercise

Structural abnormalities in cortex and midbrain associated with ADHD likely develop slowly over years. Similarly, structural changes in cortical and subcortical regions develops slowly over weeks to months of e.g., motor practice [126,127,128], and we accordingly restrict the discussion of exercise effects to long-term interventions and association between physical activity levels and structural integrity/volume. 

#### 2.1.1. ADHD Is Associated with Structural Cortical, Cerebellar and Subcortical Abnormalities

ADHD is associated with wide array of grey matter abnormalities in the brain (see [129] for a recent overview). Of great interest, a recent study found neuroanatomical correlates of ADHD to overlap with those of working memory across age-groups [130]. Magnetic resonance imaging (MRI) studies point to lower grey matter volume for anterior cingulate cortex, basal ganglia and cerebellar vermis along with lower frontal, parietal and temporal cortical thickness [53,131,132,133,134,135,136,137,138,139,140]. Evidence from diffusion-weighted MRI has indicated that the grey matter abnormalities are paralleled by differences in white matter organization in prefrontal, frontostriatal, frontoparietal and mesocorticolimbic circuits [141,142]. Intriguingly, white matter differences between structures, which are intimately involved in attention control and reward processing have been demonstrated to predict persistence of ADHD into adulthood [143]. Although it does not provide causal evidence, the finding suggests a link between the integrity of key brain structures and ADHD symptomatology, and this is further supported by findings demonstrating correlations between symptom severity and volume of reward related basal ganglia structures [144]. It is unlikely that specific focal structural abnormalities should underlie the heterogenous symptomatology of ADHD, but experiments in human and non-human primates with focal cortical lesions reveal increased distractibility as well as impaired selective and sustained attention ability, strongly implicate the right dorsolateral prefrontal [145,146,147] cortex but also the temporoparietal junction [148]. Furthermore, selective inhibition of α_2_ receptors in the prefrontal cortex of non-human primates causes hyperactivity and impairs behavioral inhibition [149,150], which provides a link from brain structure and function to behavior. The finding also emphasizes the role of monoaminergic signaling in cortical regions involved in executive functions, which is reviewed in next sections. Taken together, these findings provide a structural basis of the prevalent hypothesis that dysfunctions and delayed development of brain circuitries contribute to ADHD symptomatology, although it is important to note that the involved mechanisms may indeed not be unidirectional.

#### 2.1.2. Exercise Leads to Structural Changes in the CNS

Exercise might act as an endogenous stimulus to trigger a cascade of molecular neuroplastic processes eventually leading to structural adaptations in the nervous system (see e.g., [151] for review). Two decades ago, Van Praag and co-workers demonstrated that voluntary treadmill running led to increased neurogenesis bilaterally in the dentate gyrus of the hippocampus in rats [152,153]. These findings were later extended to also encompass morphological adaptations in areas demonstrating attenuated development in ADHD, including the prefrontal cortex (PFC) [154]. In humans, several cross-sectional studies indicate that higher fitness levels are associated with both structural and functional differences in multiple cerebral structures, which are intimately involved in cognitive functioning [90,91,93,155,156,157,158]. The modulating effects of exercise interventions on brain structure have especially been substantiated in older adults, with ample evidence suggesting that exercise is a potent strategy to mitigate atrophy of brain volume associated with aging or even lead to increased grey and white matter in frontal regions as demonstrated by Colcombe et al. [159]. In children and adolescents, only a few studies have investigated the structural differences and adaptations associated with physical activity levels and exercise, respectively. Drawing on cross-sectional evidence, aerobic fitness, an indirect marker of physical activity levels, has been associated with larger volumes of both subcortical and cortical structures, including the dorsal striatum of the basal ganglia [90]. In contrast, for neocortical structures, higher-fitness children have been demonstrated to exhibit lower grey matter thickness in superior frontal and superior temporal cortical areas [160]; two areas that typically undergo substantial grey matter pruning during adolescence [161,162]. These findings tentatively suggest that higher-fit individuals outrace their less-fit peers in developmental progress for these specific subcortical and cortical structures. Conversely, this stage of cortical development is reached with further delay in individuals with ADHD compared to individuals without ADHD [53]. Interestingly, most of the abovementioned studies linking aerobic fitness with structural differences included parental reports of the level of ADHD-related traits, but either excluded participants displaying a high degree of ADHD-related traits or failed to explore moderated associations statistically—potentially due to the relatively small and recurrent sample size included. This approach unfortunately filters out information relating to the extremes of neurocognitive and structural development, making direct inferences to ADHD affected populations troublesome.

Studies probing white matter integrity are few. A cross-sectional study in a sample of PBC children utilized diffusion-tensor imaging (DTI) to demonstrate that white matter integrity in fronto-temporal bundles is higher in individuals with higher aerobic fitness levels [94]. The results from a single longitudinal randomized controlled trial in undiagnosed children, has furthermore demonstrated that 8-months of exercise training resulted in increases in the fractional anisotropy of the uncinate fasciculus, which connects frontal and temporal areas. The results are, based on the 18 individuals enrolled in the study, indicative of greater white matter integrity in the intervention group following exercise [163,164]. In sum, results from PBC children tentatively suggest that markers of physical activity levels or exercise per se are associated with modest structural adaptations in some of the structures and networks of the nervous system displaying protracted or anomalous development in individuals diagnosed with ADHD. As these structures and networks have been related to performance within several cognitive domains including EF, it could be hypothesized that exercise-induced structural adaptations may potentially be accompanied by changes in performance in e.g., tests of EFs in children and adolescents with and without ADHD. Whether this is in fact the case, however, warrants further investigations, as the current bulk of evidence is largely cross-sectional and relies on a recurrent sample of individuals and the few longitudinal studies have largely been confined to PBC individuals.

### 2.2. Abnormalities Associated with Neuronal Network Activity in Pediatric ADHD and the Effects of Exercise

The grey and white matter deficiencies associated with ADHD are likely to contribute to the aberrant patterns of activity and connectivity observed during resting state and task-based functional MRI (fMRI) and electroencephalography (EEG) assessments [165]. Conversely, behavioral patterns may indeed also lead to neuroplastic changes in the central nervous system. In any case, it is interesting to investigate brain activity patterns in individuals with ADHD and compare this to activity in PBC individuals—also to understand the possible effects of exercise. Here, we describe networks or nodes of networks, which have been demonstrated to display aberrant activation in children with ADHD during cognitive processing. The evidence originates from noninvasive electrophysiological and brain imaging methods.

#### 2.2.1. ADHD is Associated with Altered Activity in Networks across the Brain

Engaging in meaningful sensorimotor interactions with our surroundings depends on our ability to structure brain activity patterns, which are necessary for planning, initiating and executing movements, perceiving sensory inputs and also the ability to attenuate resting-state central nervous activity characterized by default mode network activity during cognitive processing [166]. Incapacity to do so is associated with impaired attention and inhibitory control thus ADHD [167,168,169].

Hyperactivity, inattention and impulsivity in children has early on been suggested to be reflected in a cortical slowing of processing in frontal regions i.e., and increase in slow (theta, ~2–8 Hz) band activity in EEG recordings [170,171] reflecting decreased alertness possibly due to abnormal monoaminergic transmission in the corticocortical and corticostrital networks described below (but see also [172] for a review). The relative theta-to-beta (16–25 Hz) activity over midline electrodes (i.e., the theta/beta ratio, TBR) represents an FDA approved adjunct diagnostic tool for pediatric ADHD although it is associated with some controversy (see [173] for discussion and meta-analysis). In addition to the TBR, alpha band activity (7–13 Hz), a slow alpha peak frequency is commonly reported with ADHD [174,175,176], and this is thought to signify state of arousal [177] and previously demonstrated to predict psychomotor performance [178,179].

Not only EEG recordings but also evidence from fMRI has indicated differences in network activation involving frontal brain regions in ADHD and this is commonly referred to as hypofrontality. Evidence from meta-analyses suggests that hypoactivation of frontostriatal (FSN), frontoparietal (FPN) and ventral attentional (VAN) networks during inhibitory control tasks represent correlates of pediatric ADHD [180,181]. Altered activity in frontostriatal circuitries is thought to underlie several reward-related behavioral impairments [182]. ADHD is associated with hypoactivation of frontostriatal circuits during inhibitory control tasks whereas hypoactivation of structures in the mesocorticolimbic network has been demonstrated during reward anticipation [183,184]. Patients with ADHD display decreased striatal activation, which is correlated to reward anticipation [185,186] as well as a preference for smaller immediate versus larger delayed rewards and riskier reward-related behavior (see [187] for a review). This provides a plausible mechanistic background for the effect of dopamine and norepinephrine reuptake inhibitors and more importantly for the topic of the present review, also a potential neurophysiological mechanism, which physical activity and nutrition interventions may influence (see e.g., [188]). The frontoparietal network is widely distributed and includes regions within frontal, parietal, cerebellar, insular and cingulate areas, with the ventral attentional network extending between temperoparietal, insular and ventral frontal loci [189]. FPN activation is associated with executive, goal-directed processes such as combining information from surroundings with internal representations to guide decision-making. Changes within FPN may contribute to impulsive and hyperactive behavior as recently suggested by Tegelbeckers and co-workers [190]. Attentional control is governed by a ventral and a dorsal attention system located in frontoparietal areas [191]. The ventral attention system supports the ability to reorient to external salient and relevant stimuli [192] whereas the dorsal attention system draws on executive processes outlined above albeit influenced by bottom-up processes reflecting salience of external stimuli. Evidence links aberrant function in these attentional control networks to deficiencies in selective and sustained attention associated with ADHD (e.g., [193]). Furthermore, these networks along with tonic alertness are linked to monoaminergic projections from subcortical structures [194,195]. The high temporal resolution of EEG allows time-locking recordings and stimuli and thereby to study event-related potentials (ERPs) by averaging many evoked responses (see [196,197] for a review of ERP in cognitive sciences). Individuals with ADHD generally display compromised task-related neural processing [198], manifested as reduced amplitudes of ERPs e.g., the N200 and P300 component during tasks requiring executive inhibitory/interference control [198]. The reduced P300 component ostensibly reflects a compromised capacity to allocate attentional resources effectively during cognitively demanding tasks [199].

#### 2.2.2. Exercise Leads to Changes in Network Activity

Exercise has been demonstrated to change oscillatory activity in the ‘resting’ brain, but also task-related activity across several cerebral loci. Resting EEG measurements demonstrate that in young adults, acute exercise increases resting state alpha peak frequency [200] and beta power [201]. In both PBC and ADHD children, coordinative physical activity of moderate intensity (metabolic value (MET) of 4) has been found to decrease theta and increase alpha power [202], whereas treadmill running at a moderate intensity leads to nearly normalized TBR in children with ADHD [203,204]. Changes in resting state activity have been investigated with fMRI as well. In PBC adults, Weng et al. found acute exercise to increase functional connectivity within reward networks and increased integration between executive and attentional control networks as well as between dorsal and ventral attention networks [205].

Ample evidence in PBC children suggests that acute exercise can change event-related brain responses, (e.g., the amplitude and latency of the P300 component) during cognitive tasks taxing executive functions (e.g., [72,206,207]). A few reports of changes in task-related activation following acute exercise exist. In young adults, Li et al. increased task-related (2-back task) activation in prefrontal areas associated with WM functions and decreased activation of regions within the default mode network [208]. In PBC children, Chen and co-workers found increased activation superior and inferior parietal lobule along with and posterior lobule of cerebellum [209]. In further support, increments in task-related blood oxygenation in PFC after exercise measured with Functional Near-Infrared Spectroscopy (fNIRS) have been demonstrated in PBC children and adults correlating with improved performance on executive tasks (N-back [210] and Stroop [211,212,213,214]). This may reflect increases in the state of arousal, which benefits executive control measured as attentional performance [215]. In summary, an acute bout of exercise appears to affect the resting brain reflected in decreased TBR and increased α-peak frequency as well as increased functional connectivity within networks intimately associated with executive and attentional control. Parallel changes in task-related activity are evident as increased blood oxygenation assessed with both fMRI and NIRS and increased event related brain responses. Conceptually, acute exercise might thus provide an avenue to counteract the functional deficiencies observed in cognitive processes in young individuals with ADHD.

In children with ADHD, one study examined EEG-correlates of stimulus processing and attentional resource allocation following a single bout of exercise. Intriguingly, the study revealed increases in the amplitude of the P300 component both in non-medicated children with ADHD and in PBC children following 20 m of moderate intensity treadmill running compared to seated reading [78]. Furthermore, the latency was shortened over frontal regions, which is indicative of improved processing speed. A single bout of exercise might thus transiently elevate the available attentional resources and improve covert processing speed in children with ADHD and in PBC children. These results highlight that changes in task-related neural processing following acute exercise are comparable between PBC and ADHD-diagnosed individuals. This is further supported by a recent study demonstrating non-dissociable increases in P300 amplitudes following exercise in children with ADHD receiving MPH treatment and in PBC children [76]. Altogether, these findings lend credence to the hypothesis that task-related cognitive processes are equally susceptible to the influence of exercise in diagnosed and undiagnosed individuals.

In young PBC children, aerobic fitness levels have been associated with hyperactivation of prefrontal regions during an Eriksen flanker-task assessing inhibitory control [91], albeit the results are ambiguous [158], with discrepancies potentially linked to successful versus unsuccessful compensatory behavioral strategies. A handful of studies have investigated task-related neural activity based on fMRI before and following long-term exercise interventions. Davis and co-workers found that adhering to an afterschool exercise program 5 days/week for 13 weeks led to bilateral increases in task-related PFC activity during the anti-saccade paradigm in a sample of 20 overweight children aged ~10 [216] (but see also [217] for contrasting findings). For longer exercise interventions, the available results are also compelling, but less dense. One study found increased activation in the PFC following an 8-month exercise intervention in overweight, unfit PBC children [218]. Another study by Hillman and co-workers reported greater P300 amplitudes in 109 preadolescent children adhering to a 9-month afterschool exercise program compared to waitlist controls, suggesting that the beneficial effects observed following single exercise bouts might accumulate over time [219]. While these results are promising, other studies have failed to replicate and extend these results [220]. This might reflect differences in the characteristics of the interventions employed, but also heterogeneity in the populations tested in previous studies. For example, almost 50% of the children participating in the RCT set out by Hillman and co-workers were characterized as pertaining to low socio-economic status, and this might be an influential moderator of effect sizes. Nevertheless, in line with the evidence from acute behavioral studies, the results tentatively suggest that chronic exercise benefits event-related neural processing in those who need it the most and this potentially holds promise for individuals with ADHD. However, how electrophysiological processes related to cognitive functions change following both acute and chronic exercise interventions in individuals diagnosed with ADHD remains to be thoroughly elucidated. Furthermore, another major challenge for the studies applying ERP-related techniques is the lack of reports linking exercise-induced changes in event-related activity and cognitive performance (but see [221] for exceptions using cluster-based permutation techniques). The functional and clinical relevance of changes in event-related electroencephalographic activity following exercise bouts is therefore not fully understood, and changes could in principle be epiphenomena unrelated to function. In addition, resting EEG measure of the long-term effects of exercise is hard to interpret. As an example, two weeks of exercise caused increases in delta band spectral power, but decrements in all other all others along with increased mean band frequency of delta, theta and beta, but not alpha bands [222]. The long-term alterations in PBC children are thus not readily interpreted, and to the best of our knowledge, changes in resting EEG in children with ADHD after long-term exercise are yet to be been investigated. In the following section, we review neurobiological changes at a biochemical level, supporting the behavioral benefits and neurophysiological changes accompanying short and long-term exercise.

### 2.3. ADHD, Exercise and Biochemical Changes in the Brain

Here, we briefly describe experimental findings in support of the monoaminergic hypothesis of ADHD as well as findings linking pediatric ADHD to anomalous BDNF activity. Exercise is theorized to influence ongoing and subsequent cognitive performance through multiple central nervous routes such as changes in cortical monoaminergic transmission, changes in brain neurotrophin levels and changes in cerebral blood flow. Acute exercise increases cerebral oxygenation and while the Kety-Schmidt determined global cerebral blood flow remains largely unchanged during most exercise types [223], regional changes in oxygen consumption, glucose and lactate direct further blood flow to activated areas. The repeated increased metabolic demands associated with e.g., running in long-term interventions increases angiogenesis in cerebellum [224,225], motor cortex [226,227,228] and striatum [229]. The two former alterations are within motor areas and suggest compensatory angiogenesis to accommodate region-specific metabolic demands. These changes do not immediately appear relevant for effects relating to executive functions and/or behavior in ADHD. However, both motor cortical and cerebellar regional activity has been implicated in cognitive functions [228] and ADHD symptomatology [86,136,230]. Interestingly, the exercise-mediated increases in vasculature are triggered by increased neurotrophic signaling [229].

With a particular focus on dopamine (DA) and brain-derived neurotrophic factor (BDNF), we discuss the mechanisms, which are thought to be involved in mediating effects of exercise on performance in cognitive tests in children.

#### 2.3.1. ADHD is Associated with Abnormal Monoaminergic Signaling

As the ADHD affected networks and cerebral structures described above entail multiple monoaminoceptive regions [231,232], it is tempting to speculate that dysfunctional regulation of monoamines and monoaminergic signaling may represent a biochemical underpinning of the reported ADHD related patterns of brain activity.

##### Dopamine and Reward-Related Processing

It has been suggested that impaired transmission in motivational- and reward-related pathways contributes to the ADHD pathology (e.g., [31]). In support of this, and as discussed above, impaired reward processing is linked to aberrant recruitment of frontostriatal networks (see [233] for review). Specifically, children with ADHD prefer immediate smaller rewards over delayed larger rewards ([234] but see also [235] for a review). In humans, this is evident as hyporesponsiveness in striatum during reward anticipation [185]. As reward processing is intimately linked to transmission from midbrain to striatal and prefrontal regions [236], altered dopaminergic activity presents a likely candidate mechanism.

The ameliorating effects of stimulants, which are first-in-line treatment for children with ADHD, support the pivotal role of DA transmission in pediatric ADHD. The over-all effect of MPH and amphetamine is to increase extracellular catecholamine availability and whilst they differ slightly in the potency of the direct effects on monoaminergic reuptake transporters, the effects on ligand availability are comparable (see [237] for an excellent overview). MPH and amphetamines inhibits the dopamine transporter (DAT) [238] and cause increased release of DA containing vesicles [239,240]. The net result is increased extracellular DA concentrations and signaling in striatal, prefrontal, anterior cingulate cortex (ACC) as well as other cortical regions [241,242]. In summary, stimulants improve ADHD symptoms [243] and executive functions [244,245] through altered central nervous monoaminergic transmission, with increased cortical and striatal DA availability appearing as the most important mediators [246].

In further support of the dopamine hypothesis in ADHD, the spontaneous hypertensive rat (SHR), a commonly used rodent model for ADHD, is hyperactive; expresses lower ability to sustain attention and increased impulsivity in the absence of immediate rewards along with increased response variability [247,248], which can be reversed by amphetamine administration [249,250,251]. Furthermore, SHR displays differences within *DAT1* compared to their progenitor Wystar Kyoto Rats [252] and aberrant DA activity [253,254].

Despite a high hereditability of ADHD, which is estimated to ~75% for both inattentive and hyperactive components and all genders (see [255] for a recent review), only one genome-wide association study has identified 12 independent common variant risk loci that met genome-wide levels of significance [256]. These include loci associated with other psychiatric diseases in addition to the DUSP6 gene coding for the a phosphatase involved in regulation of DAT internalization and thus synaptic DA activity [257]. A meta-analysis from 2009 pointed to common variants in six genes coding for proteins involved in serotonin (5HTT, HTR1B), dopamine (DAT1, DRD4, DRD5) and SNAP-25 a protein involved in vesicle fusion with the presynaptic membrane (SNAP25) [258]. Supporting the view that ADHD represents the extreme of continuous traits, the 10-repeat allele of a variable number of tandem repeat in the intronic region of the DAT1/SL6CA3 gene predicts higher levels of inattention and hyperactivity across the general population as well as categorical diagnosis in male youth [259].

The relation between ADHD and gene variants coding for decreased bioavailability of in particular dopamine underlines the potentially beneficial role of acute exercise, since exercise can be considered an endogenous route to increased CSF (intra- and extrasynaptic) dopamine concentrations [260].

##### Control of Attention and Monoaminergic Signaling Systems

In terms of attentional control, a system of three interconnected networks with distinct neurochemical circuits subserving attentional processes was proposed early on [194,195]. These networks include a general tonic alertness network involving noradrenergic innervation from locus coeruleus and an orienting network associated with the visual system, frontal and parietal lobes and cholinergic transmission and lastly an executive network in LFPC and ACC, which is coupled with dopaminergic projections from midbrain areas. More commonly the neurophysiological correlates of attentional control are described with a 2-system model, which encompasses a ventral and a dorsal attention system located in frontoparietal areas [191]. The former supports the ability to reorient to external salient and relevant stimuli [192] whereas a dorsal attention system draws on the executive processes outlined above albeit influenced by bottom-up processes reflecting external stimuli salience. Evidence implicates both of these attentional control networks along with tonic alertness in deficiencies in selective and sustained attention associated with ADHD (e.g., [193]).

Aberrant norepinephrine (NE) signaling has been demonstrated in relation to ADHD (see e.g., [261,262]) and it is well described that NE projection activity from Locus coeruleus plays a role in vigilance and attentional processes [263,264,265]. In humans, children with ADHD have lower baseline concentrations of peripheral NE when compared to their PBC counterparts [266]. Although, the relationship between peripheral NE and central noradrenergic signaling is poorly understood, an association to tonic NE signaling mediating general alertness cannot be excluded. Supporting a role for NE in ADHD pathology, stimulants improve cognitive functions through NE mediated pathways as illustrated by increased NE signaling in striatum after AMP administration in non-human primates and rats [267] and improved sustained attention ability after administration of a selective α_2A_ agonist in children with ADHD [268].

As discussed above, candidate gene studies implicate 5-hydroxytryptalmine (5-HT) i.e., serotonergic signaling in the ADHD pathology (e.g., [269]), whereas evidence linking cholinergic transmission to ADHD related deficits in cognitive functions is sparse. The role of 5-HT signaling in ADHD is supported by clinical trials demonstrating beneficial effects of buspirone (e.g., [270]) on behavioral modulation and the effect is speculated to relate more to impulsive and hyperactive behavior rather than to inattention (see [271] for a review). Furthermore, the non-stimulant drug Atomoxine binds to both norepinephrine and serotonin transporters at clinical doses [272]. The reported improvement in sustained attention and executive functions [273] including response inhibition [274] following Atomoxetine administration can thus be due to changes in both noradrenergic and serotonergic signaling. Finally, abnormalities in dopaminergic synaptic markers within reward-associated networks incl. midbrain, caudate and accumbens have been demonstrated to correlate with symptoms of inattention, which suggest a role of dopamine and the frontostriatal executive attentional control system in ADHD [275].

#### 2.3.2. Exercise Changes Monoaminergic Signaling

The neuroendocrine response to exercise is intricate and it is beyond the scope of this review to provide a complete overview of the involved mechanisms and effects (see instead [276,277]). Here, the focus is in particular on the response of the central nervous monoamine diffuse systems. During exercise, plasma levels of catecholamines [278,279,280] increase acutely with longer lasting increments of DA fitting the temporal profile of exercise-induced improvements in cognitive functions best. Central monoamine levels are also affected by exercise, but the relation between changes in peripheral and central levels is complex. For example, CNS NE levels are more related to peripheral epinephrine than NE, DA does not cross the blood brain barrier and central changes in DA have been suggested to depend on peripheral calcium stimulating central DA synthesis [281,282]. Accordingly the finding that acute exercise-induced increases in systemic catecholamines are blunted in children with ADHD as compared to PBC children does not suggest that exercise benefit exclusively the latter, but rather that the peripheral response to exercise may entail diagnostic value [266]. In rodents, striatal and prefrontal DA and 5-HT levels increase with exercise [283,284,285,286,287] (but see also [288] for contrasting findings), whereas changes in central NE are equivocal at best. In brief, it appears that extra striatal areas are NE depleted whereas striatal [283,289] and prefrontal [290] extracellular NE activity increases. The increase in DA activity is presumably mediated through reciprocal effects on DRD1 and DRD2 receptors.

Longer lasting adaptations in the monoaminergic systems are evident after long-term exercise. Following weeks of treadmill running increased expression of tyrosine hydroxylase (TH) alongside decreased expression of DRD2 autoceptors in substantia nigra indicate increased DA synthesis and release from midbrain projections [291]. Impaired TH expression in substantia nigra [292,293] and striatum [293,294] in the SHR model is counteracted by 4 weeks of exercise, and the response to exercise is dose-dependent and coincides with reduced hyperactivity. Also in SHRs, Cho et al. demonstrated that long-term exercise counteracts hyperactivity and impulsivity through decreases in striatal and substantia nigra DRD2 expression [295]. Findings of higher central nervous levels of NE has also been demonstrated in trained versus untrained rats [296,297]. In SHRs, weeks of treadmill running leads to normalization of orienting behavior mediated through reduced norepinephrine transporter (NET) levels in PFC [298] and causes structural changes in PFC [154]. These findings emphasize that exercise has beneficial effects on cognition also through NE dependent mechanisms.

In summary, acute exercise affects the same DA and NE systems as stimulants (see e.g., [299] for review), which may mediate the beneficial effects on NCFs reviewed above. Furthermore, evidence from murine models suggests long-term exercise to normalize function of dopaminergic nigrostriatal signaling and prefrontal NE function. Moreover, recent findings suggest that the beneficial effects of exercise on memory depend on the allele composition within genes that influence dopaminergic transmission [300,301]. It remains to be investigated whether similar relations exist for cognitive functions outside of the memory domain.

It is important to mention that, the hereto described effects on monoamine signaling most likely only constitute a few of the potential avenues of which exercise may change central nervous structure and functions (see [302] for a review).

#### 2.3.3. ADHD is Associated with Abnormal BDNF Signaling

Brain-derived neurotrophic factor is expressed across the mature mammalian central nervous system in minute amounts [303]. It acts through the high-affinity receptor Trk-B, potently modulates synaptic signaling and neuroplasticity as well as influences neuronal maturation, cell differentiation, cell migration, cell proliferation and survival (see e.g., [304]). The role of BDNF signaling in declarative and procedural memory processes is well-described (e.g., [304,305,306,307]). Despite persistent suggestions of a mechanistic role [308,309,310], evidence of a BDNF contribution to ADHD core symptomatology and EFs in ADHD is less robust.

Whereas the Val^66^met single-nucleotide polymorphism (SNP) has been argued to influence EFs as well as related neural structures in late adulthood (e.g., [311]), meta-analyses demonstrate that it does not impact executive functioning [312] or increase risk of ADHD in children [258,313]. Recent findings do however suggest that the Met allele (generally associated with lower BDNF activity and impaired declarative memory functions [307]), is associated with larger cortical area and thickness of parietal and prefrontal cortices in children [314,315], but also with hyperactivity and impulsivity [316]. Investigations of associations between ADHD, non-memory related cognitive functions and the Val^66^Met SNP have resulted in contrasting findings at best. This does not preclude the possibility that variation within the BDNF gene may be involved in ADHD pathogenesis, but it suggests that this is not mediated by the Val^66^Met SNP alone but rather a haplotype, which includes this locus [317] (but see also [318] for an overview) or rare gene variants [319].

Systemic BDNF (i.e., serum and/or plasma) concentrations relate to central nervous levels as demonstrated by highly correlated ontogenic changes in measures of frontal cortex and serum BDNF concentration [320]. Findings of both similar, lower and higher systemic BDNF levels in ADHD patients compared to PBC children and adults exists (e.g., [321,322,323,324]) and a recent meta-analysis found similar levels of BDNF in children but with males displaying increased systemic BDNF [325]. Reported increases in plasma BDNF coinciding with reduced hyperactivity following long-term MPH treatment could suggest a compensatory function [326].

The interaction between mechanisms involved in BDNF and dopamine signaling provide a likely route of which exercise-induced secretion of BDNF may exerts effects, which can influence executive functions [327]. Despite the lack of BDNF mRNA in murine striatum [328] the BDNF protein is widely distributed due to anterograde axonal transport from several loci including substantia nigra, cortex, thalamus and amygdala [328,329]. Also, the high affinity BDNF receptor Trk-B expressed neurons [330] are vastly present in basal forebrain and striatum [331]. In striatum, presynaptic binding of BDNF to its high-affinity receptor Trk B increases the release of DA alongside 5-HT and GABA [332,333], and striatal infusion of BDNF improves executive function (set-shifting) through Trk-B mediated mechanisms with an inverted u shaped dose-response curve although this effect is proposed to depend on potentiation of glutamate release [334]. Acute AMP administration increases BDNF expression in striatum and ACC in wild-type mice, but not in the heterozygous BDNF(^+/-^) model with only one functional allele [335]. Furthermore, only wild-type mice display increased midbrain TH expression further linking BDNF to DA signaling.

In summary, in contrast to the monoamines, genetic differences in the *BDNF* gene do not affect NCF in children with ADHD. Changes in peripheral BDNF may signify increased central nervous BDNF activity and likely influence DA transmission through its high affinity Trk-B in PFC and striatum. The latter provides a likely route of which BDNF may mediate the beneficial effects of exercise on NCFs.

#### 2.3.4. Exercise Changes BDNF Signaling

In this section we discuss effect of exercise on neurotrophic factors in relation to ADHD limited to BDNF. The effects of aerobic exercise on BDNF expression and functions of the medial temporal lobe, which are underlying the enhancing effects on (declarative) memory has been reviewed in depth previously (see e.g., [24]). As ADHD is not associated with impaired long-term episodic memory, we instead focus on BDNF transmission outside of hippocampus and the effect of non-memory related neurophysiological and cognitive processes [336]. A recent meta-analysis concluded that acute exercise regardless of exercise modality increases peripheral measures of BDNF (i.e., plasma or serum BDNF) [337]. Importantly, both circulating and central levels of mature BDNF increases after acute moderate to vigorous exercise with cortex and hippocampus as the main sources [338] although the ability of BDNF to cross the blood-brain barrier remains somewhat controversial [339,340,341,342]. In rodents, days to weeks of exercise affect expression of BDNF in cerebellum, frontal cortex and striatum alongside increased phospo-Trk-B in striatum [343,344].

In humans, the BDNF Val66Met SNP has been demonstrated to mediate the relationship between levels of physical activity and working memory [327]. Furthermore, reports of parallel exercise-mediated increments in circulating BDNF and performance in working-memory (Digit-span) [345], cognitive-flexibility (task-switching) [346], attention (visuospatial attention task) [347], working memory + attention (Wisconsin Card Sorting Test) [348], selective attention + behavioral inhibition (Stroop) [349,350] (although a null-finding on the Stroop exists [351]) tasks suggest that BDNF plays a role in mediating effects of exercise on NCFs. A causative role remains, however, to be demonstrated.

## 3. Exploring the Parameter-Space of Exercise Characteristics: Effects of Exercise on Cognitive Performance in Children and Adolescents with ADHD

Since exercise may be structured in numerous different ways with respect to duration, intensity, total volume and activity type (modality) etc., these parameters naturally also differ between the existing studies within the research area. In order to understand the potential effects of exercise on cognitive performance in children and adolescents with ADHD, it is thus important to elucidate if observed effects relate to specific parameters of both acute and long-term exercise. In the analysis performed in the present review, we have identified the existing studies on effects of acute and long-term exercise on cognitive performance in children and adolescents with ADHD and quantified effects on performance within specific cognitive domains and relating to the exercise parameters duration, intensity and volume (see Appendix A for details on systematic search, description of exercise parameters and calculation of ES). Beneficial effects of physical activity on cognition are contingent on the exertion (i.e., an element of exercise, and not mere activity or movement per se) [70]. Accordingly, we have not included activities such as recreational park walks [352], horse-back riding [353] or yoga [354,355] in the analysis as potential effects of meditation alongside with exposure to nature, animals etc. are beyond the scope of this review. The analysis of the existing studies within the area allows us to consider and discuss which aspects of exercise contribute to any eventual derived effects e.g., on performance in neuropsychological tests. This discussion is important both from a mechanistic point of view, but also in an applied perspective and thus coherent with the purpose of the present review.

**Figure 2:**
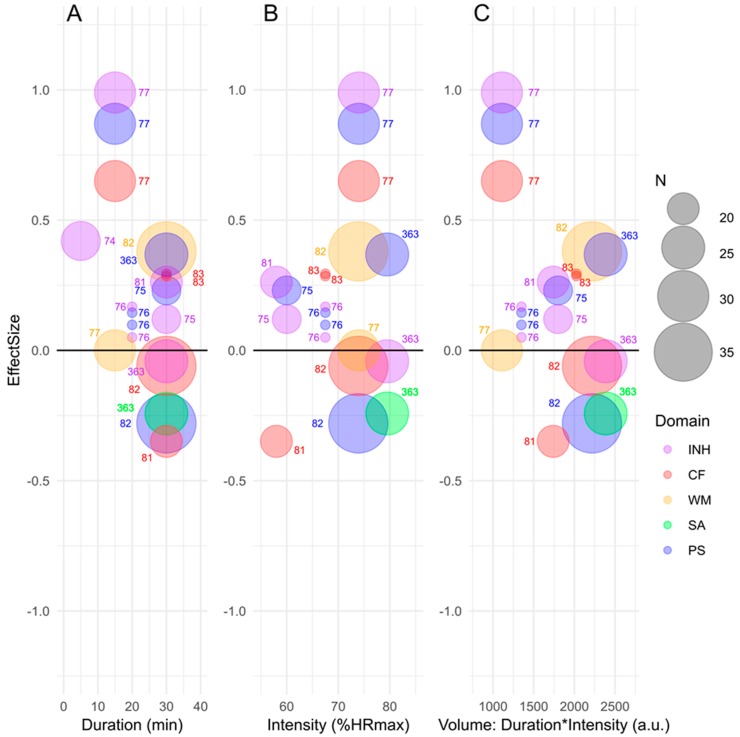
The effects of acute exercise on cognitive performance for children with ADHD. The three parts of the figure depict effect sizes extracted from studies systematically identified and categorized by cognitive domain (inhibitory Control (INH, purple); Cognitive Flexibility (CF, red); Working Memory (WM; orange), Sustained Attention (SA, green) and Psychomotor Speed (PS, blue) as a function of exercise intensity (**A**), duration (**B**) and volume (intensity x duration, (**C**)). The size of the circles denotes the number children with ADHD (within subject design) allocated to the active group or groups (between subject design) that completed the study.

Figure 2 depicts the effects (ES) of acute exercise on cognitive performance for children with ADHD and the effects are specified for exercise duration, intensity and volume for the cognitive domains inhibitory control, cognitive flexibility, working memory, sustained attention and psychomotor speed. Based on Figure 2 the studies investigating acute exercise find small to moderate beneficial effects of acute aerobic exercise on executive functions in children with ADHD.

**Figure 3:**
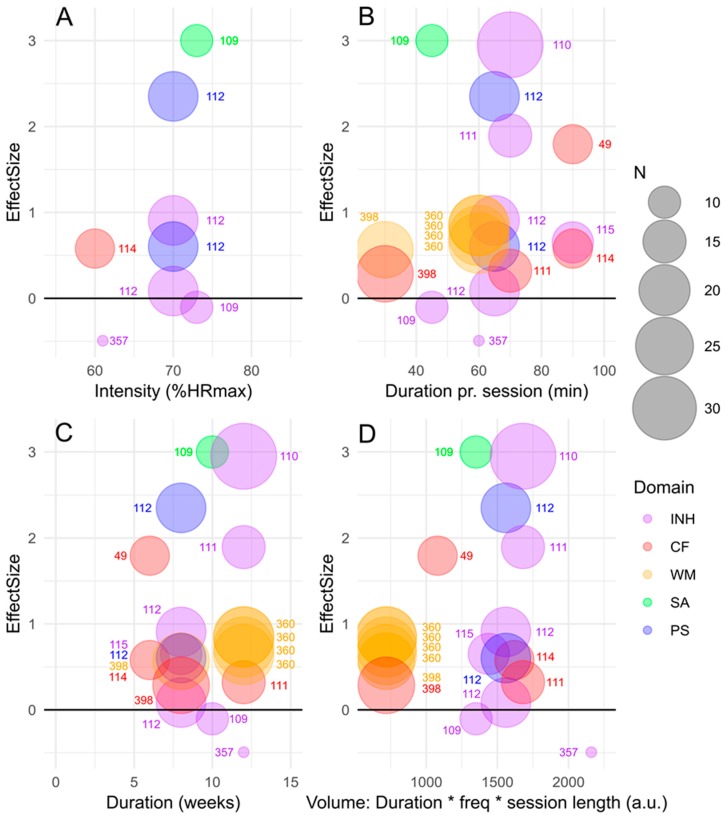
The effects of long-term exercise on cognitive performance for children with ADHD. The multi-plot illustrates extracted effect sizes color-coded by cognitive domain and plotted against exercise intensity (**A**), session duration (**B**), study duration (**C**) and volume (session-duration x session frequency x study duration, (**D**)).

Figure 3 depicts effects (ES) of long-term exercise on cognitive performance for children with ADHD and the effects are specified for exercise intensity, duration pr. session, duration of the intervention and exercise volume. Also, for the effects of long-term exercise, the effects are specified for the cognitive domains inhibitory control, cognitive flexibility, working memory, sustained attention and Psychomotor speed. The extracted effect sizes from long-term interventions suggest consistent positive effects on cognitive performance in children and adolescents with ADHD, and it should also be noted that the effects of long-term exercise (Figure 3) appear larger than those observed following acute exercise (Figure 2).

**Figure 4:**
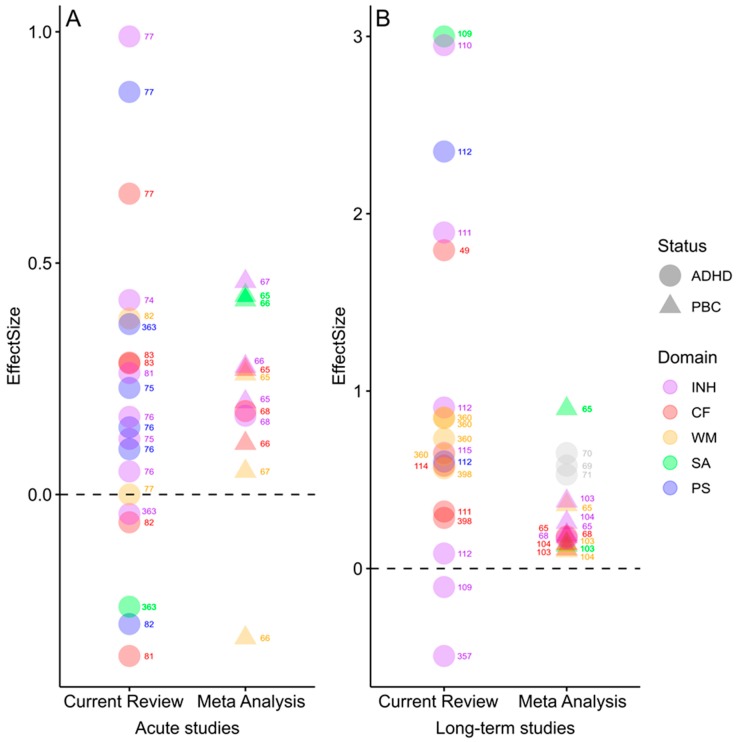
The effects exercise on cognitive performance for children with and without ADHD. Dual-plot presenting extracted effect sizes for acute (**A**) and long-term (**B**) exercise interventions color-coded by cognitive domain and plotted next to effects sizes derived from published meta-analyses in children with (circles) and without (triangles) ADHD. Effects sizes across executive functions are depicted in grey. Please, note that scale of the ordinate differ between the two plots.

In order to allow a direct comparison between the analysis performed in the present review and the results obtained in previous meta-analyses, Figure 4 depicts effects (ES) of acute (left) and long-term exercise (right) on performance in different cognitive domains for children with ADHD and population-based controls (PBC). Despite large heterogeneity between studies, previous meta-analyses find small positive effects of exercise in population-based control (PBC) children, and the results obtained in children with ADHD are coherent with this finding. In children and adolescents with ADHD there are even larger differences in ES between studies, but the results confirm the overall positive effects on cognitive performance - in particular for long-term exercise.

### 3.1. Effects of Acute vs. Long-Term Exercise on Cognitive Functions

As specified above, we have extracted effects sizes for the available studies of acute and long-term exercise interventions on cognitive performance in children with ADHD and provided a graphical overview of the effects in Figure 2 and Figure 3. Qualitatively, the results presented in Figure 2 suggest that average intensities between 65 and 75% of maximal heart rate with durations below 20 m may result in the largest acute effects. However, this should be interpreted in light of the very uneven distribution of exercise characteristics across the two parameters. The vast majority of the studies are conducted with ~75% HRmax for 30 m (incl. warm-up).

In ADHD, abnormal cortical monoaminergic neurotransmission affecting attention and reward processing has been observed. With acute exercise of moderate to high intensity however, plasma levels of catecholamines increase with longer lasting increments of DA fitting the time course of exercise-induced improvements in cognitive functions. Central monoaminergic concentrations also increase with acute exercise, and although the relation between changes in peripheral and central levels is complex, it could be speculated that acute exercise-induced changes in monoaminergic neurotransmission may contribute to the effects of acute exercise observed in individuals with ADHD.

Considering network activation, evidence from both EEG and fMRI has indicated differences in frontal brain regions in children with ADHD compared to PBC and hypoactivation of frontostriatal (FSN), frontoparietal (FPN) and ventral attentional (VAN) networks during inhibitory control tasks etc. In both PBC and ADHD children, acute exercise with moderate intensity leads to changes in network activity and furthermore moderate intensity exercise can lead to increased functional connectivity within networks intimately associated with executive and attentional control. Acute exercise may thus provide an avenue to counteract the functional deficiencies observed in cognitive processes in children with ADHD and we thus expected to find positive effects of primarily moderate intensity exercise.

In general, the computed effect-sizes appear lower in comparison to those reported in the published meta-analyses of exercise in ADHD (see Figure 4). This may partly be explained by the fact that we calculated ESs based solely on post-intervention performance alone (i.e., comparisons between tests of NCFs conducted after and exercise or control intervention). This approach was chosen to enable comparisons of ESs between studies that did not conduct baseline testing and those that did as well as to prevent false positives from ‘catch-up effects’ due to lower baseline performance in the exercise group (see [356] for a discussion of this).

As illustrated in Figure 3, this review found consistent and large positive effects sizes for long-term exercise interventions. While effects of long-term interventions could be considered as repeated bouts of acute exercise, an additional important difference between studies, is that while studies of acute exercise have often considered timing of exercise relative to assessment of cognitive performance. This is most often not considered in long-term studies. It is noteworthy that long-term exercise is accompanied by larger effects on cognitive performance and future studies could consider combining acute and long-term perspectives. ADHD is associated with a wide array of gray matter abnormalities in the brain. These structural abnormalities likely develop over weeks to months to years. Conversely, results from PBC children tentatively suggest that regular exercise during several (eight) months can be accompanied by modest structural adaptations in some of the structures and networks of the nervous system displaying anomalous development in individuals diagnosed with ADHD. Exercise thus holds the potential to induce or promote structural changes in the CNS and these effects would only be expected in long-term studies. It is positive that the present review found consistent and large positive effects for long-term exercise interventions and it could be hypothesized that long-term changes in performance in e.g., tests of EFs in children and adolescents with and without ADHD may relate to accompanying exercise-induced structural adaptations. This potential relation between changes in cognitive performance and CNS structure and function with long-term exercise however remains to be elucidated by future long-term studies.

### 3.2. Effects of Exercise on Performance in Specific Cognitive Domains

In line with our previous discussion of potential neurophysiological mechanisms underlying the effects of exercise on ADHD, we expected that cognitive functions supported by frontostriatal, dopaminergic and general tonic monoaminergic transmission would display the largest ESs for acute and long-term interventions respectively. As depicted in Figure 2, Figure 3 and Figure 4, we found positive ES in seven of nine measures of psychomotor speed (blue) and sustained attention (green), descriptively confirming our hypothesis. It should be noted that changes in PS should be interpreted with caution since increased RT (resulting in decreased psychomotor speed) may partly reflect decreased impulsivity. The ESs for acute exercise on inhibitory control are widespread. This measure reflects the ability to inhibit impulsive behavior and can thus at least in part be argued to reflect reward processing. As improvements in aberrant reward processing do not occur with acute exercise but are more likely to be reflected in slow evolving adaptations in frontostriatal networks, this finding is to be expected. In support, positive ESs were computed for five of seven measures of inhibitory control after long-term exercise with the negative ES stemming from the small populations tested by Lee et al., [357] and Verret et al., [109]. It should be noted that Lee and et al., tasked inhibitory control with the Golden’s Stroop test. As discussed in Section 1.1, the Stroop Interference score does not differ between children with ADHD and PCT children. Noteworthy, the Interference scores reported by Lee et al., were smaller than expected from the age group already at baseline, which might render this measure insensitive to detect potential exercise-induced performance increments [358]. Furthermore, Verret et al., deployed the Test for Everyday Attention for Children battery and probed inhibitory control with the ‘walk/don’t walk’ task [109]. As the task entails holding a motor response past the first ~200 ms of an auditory stimulus until it reveals either ‘walk’ or ‘don’t walk’ characteristics, we categorized it in agreement with Verret et al. It should be noted that the walk/don’t walk test is an adaption of the sustained attention to response task (SART) commonly used to test sustained attention (e.g., [358]). Re-categorizing this ES to ‘sustained attention ‘efficiently annuls the contrast to the other inhibitory control (‘INH’) EF but is in dire contrast to the very large ES of sustained attention reported from the same study based on the ‘Score pondering’ test. It should be noted that whereas Verret et al., adjust post test scores for differences in pre-test means they do not report the latter. Accordingly, it cannot be rejected that the difference between the two EF may arise from large group differences at baseline.

The effect of acute exercise on cognitive flexibility is largely positive (two of five ES < 0). In contrast to Chang et al., who did not find meta-analytic evidence of effects of acute exercise on PBC children’s performance in the *alternate use* task (categorized as cognitive flexibility), we find small positive effects (0.28 to 0.29) on two of two measures extracted from Ludyga et al. also using the alternate use task [83]. However, as Ludyga et al. contrasted post-exercise performance without performance baseline assessments, this approach is not sensitive to day-to-day fluctuations in performance, which may influence the results. From Benzing and co-workers we extracted large positive effects in the temporal domain evident as reduced RT switch costs using a modified Flanker (g = 0.66) test whereas ESs from Hung et al. were negligible but negative in the temporal domain (Task Switch, g = −0.06) [77,82]. Also, we extracted negative effects from Chang et al. (Wisconsin Card Sorting Task, g = −0.35) [81].

We extracted ESs from four studies all pointing to performance enhancing effects of long-term exercise on tests of cognitive flexibility [49,111,114]. The very large positive ES (TMT-part B, g = 1.79) for cognitive flexibility computed from Kang et al. following six weeks of mixed sports therapy stands out. We categorized the TMT –B as a cognitive flexibility based on previously demonstrated correlations between the performance on this task and perseverative errors on the WCST an oft-cited operationalization of cognitive flexibility [359], but both spatial attention, processing/psychomotor speed and motor acuity could influence the performance. Both Pan et al. and Choi et al. tested cognitive flexibility as perseverative errors on the WCST yielding small to medium ESs in our analysis (g = 0.32 and 0.58). In summary, both acute but in particular weeks of exercise appear to benefit cognitive flexibility in children with ADHD.

Across both acute and long-term studies Tan et al., previously found similar effects of exercise on inhibition and set-shifting (r = 0.17 and 0.18, respectively) although only inhibition reached statistical significance. Cornelius et al., Vysniauske et al., and Cerrillo-Urbina et al., did not report measures for the various EF domains individually, but reported larger standard mean differences for attention over EF [69] and moderate significant (g = 0.54) [71] and moderate but non-significant effects of both EF and attention respectively (g = 0.65 and 0.46) [70]. Meta-analyses based on studies in PBC children are better powered and may, based on this, be more informative. For acute effects, Chang et al. found small to moderate positive effects of exercise on cognitive flexibility (alternate use test, d = 0.11) and inhibitory (Stroop, d = 0.25 and Flanker Incongruent d = 0.29), whereas a negative (d = −0.31) was found for working memory (digit span backwards) [66]. The latter is agreement with our computed null-effect (Colour span backwards [77] and negative effect (task switch, increase global switch accuracy cost [82]). This is in noteworthy contrast to the long-term effect, we computed for digit span index score (including digit span backwards) of g = −0.64 [360]. In general, the meta-analyses suggest that acute exercise have small effects on working memory (g = 0.28 [65] and 0.05 [67]), moderate effects on attention (g = 0.43 [65] and d= 0.42 [66]) and inhibitory control (g = 0.20 [65] and 0.46 [67]). Long-term exercise has small to moderate effects on inhibitory control (g = 0.19 [65], 0.38 [103] and 0.26 [104]) and working memory (g = 0.1 [104], 0.14 [103] and 0.36 [65]) and small effects on cognitive flexibility (g = 0.18 [65] and 0.14 [103,104]). In contrast, rather divergent ESs have been reported for attention (g = 0.13 [103] and 0.9 [65]). In Figure 4, the effects reported by previous meta-analyses are depicted alongside the results of the present review.

### 3.3. Exercise Intensity, Duration and Volume as Potential Moderators of Effects on Cognitive Functions

Whereas previous meta-analyses have refrained from concluding on quantitative exercise parameters as potential moderators of effects on cognitive functions, the analysis of the present review also detail effects relating to the specified exercise parameters intensity, duration and volume. Tan et al. did not find type or number and duration of sessions to moderate effects in studies including both individuals with ADHD and autism spectrum disorder [68]. Interestingly Vysniauske et al. found larger effect sizes for longer durations of exercise, but no moderating effects from exercise intensity [71]. However, since both acute and long-term studies were included in this analysis, the results should be interpreted with caution. The literature also offers more descriptive inferences based on systematic reviews. Den Heijer et al. suggest that 30 m of individually adapted daily exercise [361] represents an appropriate duration and frequency. This suggestion is supported by Neudecker et al. who refrained from concluding on other parameters due to the paucity of studies in this area [124]. Grassman et al. suggested 30 m to improve EFs [362] and this is supported by Suarez-Manzano et al. who provided 20–30 m of moderate intensity (40–75%) for acute and >5 weeks of at least three days a week with >40% intensity for long-term exercise to improve cogntion [123]. This finding on long-term improvements in measures of cognitive functions is also supported by Haperin et al. [121]. These details on exercise intensity and duration represent suggestions based on the literature as existing evidence is yet too insufficient to substantiate such recommendations.

In studies of PBC and ADHD children alike, reports of the intended and actual intensity of the exercise intervention are frequently omitted (e.g., [49,74,110,111,360] in our analysis) and other studies report average heart rate during the exercise bouts but fail to normalize these measurements to individual maximum heart rate (e.g., [82,363]). This compromises the possibility of investigating a potential moderating role of exercise intensity in the meta analyses (e.g., [67]). Nevertheless, Chang et al. found that performance in test of cognitive function (in general) performed immediately after a bout of exercise was only increased when the exercise was of moderate or low intensity [66]. Additionally, when cognitive performance was tested almost immediately after exercise (short delay), only exercise with very light to moderate positively affected cognitive performance. If a 20 min. break in-between exercise and cognitive testing procedures was introduced, also intense exercise had positive effects on performance in cognitive tests. Thus, there is a positive effect of exercise on cognitive performance, but the effects are related to interactions between exercise intensity and timing

When considering the potential effects of exercise duration, Chang et al., pointed to a minimum duration of 11 m to elicit positive effects, whereas de Greeff and coworkers did not find any moderating effects (beta = 0.001, *p* > 0.9) [65]. For chronic exercise neither de Greeff et al., nor Alvarez-Bueno et al., found study duration to moderate the effects (B between −0.1 and 0.01 and *p* > 0.2), but Xue et al. found that exercise session durations above 90 m and interventions of >5 weeks resulted in larger effects.

In this review, we hereto refrained from discussing potential interactions between medication and exercise. In brief, whereas subgroup analyses have generally not found medication status to moderate effects of exercise on cognition (e.g., [363]), the similarities between the central nervous effects of stimulants and exercise suggest that they may interact. In support of this, Choi and co-workers found additive effects of MHP and three weekly 90 m exercise sessions over the course of six weeks on cognitive flexibility [114]. This supports the role of monoamine signalling in the ameliorating effects of exercise and suggests that exercise can be adjunct to pharmacological treatment.

### 3.4. Type of Exercise—Modality—as Potential Moderator of Effects on Cognitive Functions

A noteworthy controversy regards the possible role of the type of physical activity (see [63,356,364] for discussions). As outlined previously, the very definition of physical activity renders it multifaceted, and encompassing a wide array of different activity types in different domains, with different requirements for physical exertion, motor coordination, decision-making, social interaction etc. The body of research literature in ADHD includes activities such as treadmill running and walking, trampoline jumping, water gymnastics, track and field as well as an array of different ballgames. In brief, the vast majority of the acute exercise interventions employ relatively simple, non-coordinative exercise activities (i.e., treadmill running or ergometer biking), whereas all of the long-term interventions entail coordinative exercise activities. Due to this difference between acute and long-term studies—which is important to note—exercise modality is not illustrated as part of Figure 2, Figure 3 and Figure 4.

While the physiological response may differ a lot between various types of physical activities so could the psychosocial and cognitive demands inherent in the activities [356,365,366]. In line with this notion, it has been speculated that ‘simple’ aerobic or cardiovascular physical activities (e.g., running, biking) may have little impact on measures of executive functions, whereas the cognitive demands of other activities or sports could proposedly lead to increased performance in tests gauging executive functions [356]. A systematic review by Ng and coworkers recently found that the largest intervention effects of exercise on cognitive, behavioral and physical parameters in children with ADHD were reported for mixed exercise programs over pure aerobic exercise programs [367]. The moderating effect of qualitative aspects (e.g., the coordinative and cognitive demands) of the exercise intervention has been studied predominantly in PBC children in both acute and chronic designs. For acute exercise, Budde and co-workers utilized a between-subject design to examine whether coordinative exercise and aerobic exercise led to different changes in cognitive performance and found greater effects for the coordinative exercise [215]. Other studies have, however not found similar results [366,368], but it should also be noted that when studying effects of different activity types, these may differ in several aspects including also exercise intensity, which complicates comparison. Findings from studies of long-term exercise interventions in both laborious and in ecological settings are also heterogeneous. Koutsandréou and co-workers reported improved performance in a Letter Digit Span task assessing working memory after completing a 10-week exercise program including both cardio-vascular and motor-demanding exercise program, but with the latter improving working memory the most [369]. This supports the tenet that the motor challenges might drive improvements in working memory, in a manner similar to the neuroplasticity-enhancing effects observed in the motor system [370,371]. However, children in the motor-demanding program had a mean heart rate of 125 BPM during the motor exercises, which is lower than the cardio-vascular group (139 BPM) but substantially above resting rates (e.g., 79 BPM in the control group). While it may be that motor-demanding exercises lead to larger improvements in WM compared to cardio-vascular exercise, the potential role of differences in exercise intensity cannot be rejected. Notwithstanding, the results are in agreement with previous reports (e.g., [372]) whereas a later study has failed to demonstrate larger effects for coordinative, motor-demanding exercise [220].

The effect of exercise modality has been sparsely studied in children with ADHD. Comparing the effects of a single session of either aerobic exercise (biking) or coordinative exercise (e.g., balancing on exercise balls, one-legged stands during catch-and-throw), Ludyga and co-workers reported benefits of both compared to seated rest on general reaction time in a modified flanker task, but slightly superior effects for the biking exercise group compared to the group performing coordinative exercise [76].

To sum up, meta-analyses have addressed the controversy at hand. In children with ADHD, Cornelius and coworkers found positive effects of PA involving an imminent cardiovascular element (‘aerobic’ PA) [70]. In support, Cerrillo-Urbina et al., found evidence favoring effects of aerobic exercise on executive functions and core symptoms over yoga or physical education-based interventions [69]. For PBC children, a recent meta-analysis found that in contrast to pure aerobic exercise, a single bout of cognitively engaging exercise failed to benefit cognitive functions, whereas larger ESs were found for chronic intervention deploying cognitively engaging versus pure aerobic exercise (g = 0.29 vs. g = 0.53) [65]. For acute effects, a meta-regression analysis found better performance after ergometer cycling vs running [373]. For chronic interventions, Alvarez-Bueno et al. conducted sub group analyses and found that qualitatively enriched and quantitatively enhanced exercise benefitted different cognitive domains [103].

Below we provide recommendations for continued research in the field of exercise and ADHD, while summarizing evidence into preliminary points for real-world application of the different findings, which have been discussed so far.

### 3.5. Recommendations

Existing meta-analyses have refrained from providing recommendations, but a few studies have addressed the question of who benefits from exercise. A positive meta-relation (beta 0.03, *p* < 0.05) reported by Xue et al. between body mass index and effects on EF suggests that less fit children and adolescents may benefit more from exercise [104]. To follow up on this, it is noteworthy that individuals with ADHD are often sedentary. While the effects of exercise on EF are moderated by baseline fitness, evidence for moderating effects of age is mixed [67,97,103,104]. Even descriptive conclusions based on these meta-analyses are difficult, which leads us to suggest that exercise benefits children of all ages by interacting with the maturing nervous system both off-setting and changes the developmental trajectory of impairment positively.

#### 3.5.1. Science and Evidence-Based Recommendations

Based on the quality and heterogeneity on experimental studies, an expert panel recently concluded that evidence was insufficient to recommend exercise to increase cognitive functions in PBC children [374]. Combing evidence from PBC children and children with ADHD combined with non-existing reports of adverse effects of exercise interventions we carefully dare to disagree with the panel. Descriptively, we argue that effects depicted in figures and reviewed above suggest long-term exercise as a valuable adjunct to other treatment for children with ADHD to enhance in particular inhibitory and attentional control. The effects of acute exercise appear less robust. Of great interest, Chang et al., found that only exercise carried out during the first half of the day yielded positive outcomes [66]. This underlines the importance of timing exercise. This is supported by Hart who found that 15 min of exercise provided at the beginning of the day can reduce behaviors associated with ADHD and that while this effect dissipates over time, a short bout of 3–5 min. moderate-to-vigorous physical activity 90 m after the initial exercise, can maintain the benefits [375]. Aside from the long- term effects of exercise on neural functions supporting executive functions and attentional control, the beneficial effects of acute exercise on not only subsequent cognitive activity but also recently encoded non-declarative memory (e.g., [376,377,378,379]) underline the importance of planning exercise in close temporal proximity to cognitive challenging tasks.

Furthermore, it seems that the type of activity employed should be carefully considered. Long-term interventions appear to have largest effects when they entail both cardiovascular and coordinative elements. Such playful but exerting activities are likely to be more practical feasible and associated with higher compliance. Importantly, the physiological and cognitive load associated with different activities is inherently individual. This feeds into the idea of individualizing physical activities to match the physical and cognitive level of the involved children, so that each individual is optimally stimulated and engaged by the employed interventions (Pesce et al. [380]). Furthermore, Cook and co-workers suggest that the deficits in executive functions associated with ADHD might decrease adherence to an exercise regime in young individuals underlining the importance of motivational activities [98].

#### 3.5.2. Directions for Future Research

##### Reporting Exercise Characteristics

As evident from the fewer circles in Figure 3A compared to the rest, intensity of long-term interventions is not routinely reported. To promote the understanding of which parameters that are important for the effects and identify underlying mechanisms of action, not only average heart rates, but also the range and temporal profiles enabling analysis of time spent in different heart rate zones is needed. In addition, given the potential challenge related to exercise intervention adherence in children and youth with ADHD, it is important to report adherence rate to enable rightful interpretations of potential effects.

##### Study Design

Exercise interventions are inherently limited to single-blinded assessments. To increase quality of studies, attempts to blind participants to the intervention should be routinely implemented (see e.g., for attempt to blind parents to the intervention [381]). This is however not the case in a number of studies, where symptomatology is routinely evaluated by teachers and parents, who are not blinded to the intervention. The latter can be accommodated by evaluating the effect of exercise on standardized automated tests of NCFs.

In line with this, it is essential to design appropriate control conditions that allow careful teasing apart of factors associated with potential performance enhancing effects. A large part of the studies conducted in individuals with ADHD utilize resting, passive control conditions that entail no specific change in environment or expectancy of changes in performance. As such, for passive control conditions it remains uncertain whether effects of exercise simply result from being presented with a novel situation or being disposed to a novel environment i.e., the Hawthorne effect. Active control conditions might be suitable to overcome this limitation but need to be carefully designed to control for the factors not being put under the microscope, e.g., matching social interactions or cognitive engagement. Accordingly, effects of exercise interventions ought to be interpreted in relation to the included control conditions. In addition, the improvements in tests of NCFs for resting control groups upon repeated task-exposure (e.g., [81]) underline the importance of thorough test familiarization. Furthermore, only assessing performance only after an intervention carries challenges given that it is not possible to assess within-subject variations in baseline performance. Furthermore, it is evident from the descriptive visual summary of studies provided in this narrative, that a majority of studies have been based on a rather small sample size. Albeit perhaps a daunting quest, future studies should strive to recruit larger samples to allow stronger conclusions.

Based on the findings from the previous intervention studies and meta-analyses, future studies can additionally explore whether long-term effects are contingent on accumulation of acute effects on ongoing and subsequent cognitive processes or rather reflect the structural and neurophysiological characteristics associated with e.g., higher fitness levels. Finally, future studies should explore the ecological validity of different exercise activities and parameters relating to practice structure, activity type, timing etc. by implementing various models of these e.g., during and after schooldays etc.

##### Outcome Measures

The incomplete and inconsistent reporting of outcome measures hampers interpretation of the effects of exercise. The construct validity of test of NCFs is contingent on appropriate analysis. Pooling accuracy and reaction time scores (e.g., for Stroop or Flanker) for different task-conditions (e.g., congruent and incongruent trials) or only reporting one of them decreases specificity and occludes potential speed-accuracy trade-offs. As an example of the former, Pontifex and co-workers investigated the effects of a single bout of treadmill exercise in children with and without ADHD. The authors found increased response speed and accuracy following 20-min exercise, but findings were reported across stimuli compatibility, thus reflecting overall enhancement of neurocognitive performance. However, specific effects on inhibitory control, usually expressed as an interference score computed by differences between incongruent and congruent response speeds and accuracies in a Flanker task was not formally tested. This complicates inferences relating to the effects of exercise on performance in specific cognitive domains, e.g., inhibitory control, and effects reported on such ought to be interpreted cautiously.

## 4. Conclusions and Perspectives

In line with previous research, we find that exercise benefits executive functions and attentional control in children with ADHD. The beneficial effects are comparable to those reported in PBC with substantial and consistent improvement on test of several cognitive functions following particularly long-term exercise. An important notion is also that no study has reported negative or adverse effects of exercise. Outside of the neurocognitive realm and thus the scope of this review, exercise has been reported to, not surprisingly, increase cardiovascular fitness [382] and motor functions [109,111,360,382,383,384,385], but also to improve measures of anxiety and depression related behavior [385,386], social problems [109] and self-esteem [382] in children with ADHD. We have, broadly speaking, ignored these changes to focus the present review but we are not ignorant of their importance. Furthermore, and of great importance, exercise has been repeatedly reported to improve ADHD-related behavior (e.g., [109,113,114,115,116,381,382,385,387,388]), although null-findings exist (e.g., [389,390,391]). This highlights exercise as a low-cost, multilateral approach that, if deliberately designed and delivered, could be used in adjunct with traditional pharmacological, psychological and pedagogical intervention strategies to promote cognitive performance in children and adolescents with ADHD.

We end this narrative with a final note to stress the importance of exercise in children and youth with ADHD. Two reports of special interest support the findings by Åberg et al., [392] and Tandon et al., [393] discussed above and warrant further discussion. In a large sample size (*n* = 1615) from the general adult German population, retrospective assessment of childhood PA levels and ADHD symptom severity along with current (adult) ADHD symptoms revealed that excessive childhood exercise was associated with not carrying ADHD symptoms into adulthood [394]. In support, Rommel et al. reported that level of PA during late adolescence to be inversely related to severity of symptoms of inattention and hyperactivity/impulsivity in early adulthood [395]. These associations, albeit not experimentally proven to be causal, the results presented in this review along with the dire consequences of carrying an ADHD diagnosis into adulthood strongly suggest regular exercise to children and adolescent with ADHD.

## Figures and Tables

**Figure A1:**
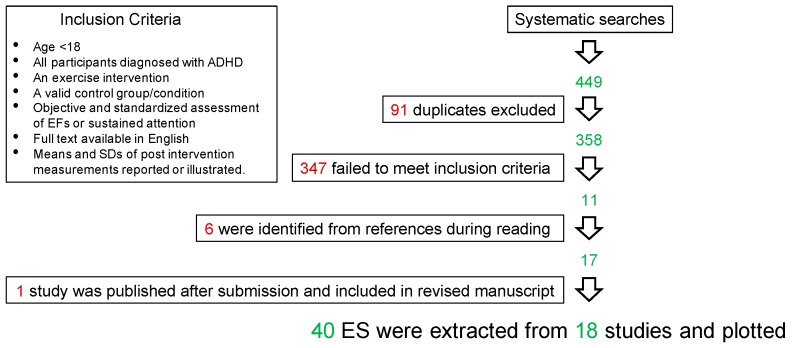
Flow-chart of the study selection process.

## Appendix A

### Systematic Search and Extraction of Effect Sizes from Previous Studies

To qualify the present discussion, we conducted a systematic search *in* relevant databases (PubMed, and Web of Science incl. Web of Science Core Collection, BIOSIS Previews, MEDLINE^®^, KCI-Korean Journal Database, Russian Science Citation Index, SciELO Citation Index, Derwent Innovations Index, Data Citation Index, Current Contents Connect) with customized search terms for PubMed:
(“exercise” [Title] OR “physical activity” [Title] OR “fitness” [Title] OR “physical exercise” [Title] OR “acute exercise” [Title] OR “chronic exercise” [Title] OR “aerobic” [Title] OR “resistance” [Title] OR “anaerobic” [Title] OR ”coordinative” OR “training”) AND (“attention deficit hyperactivity disorder” [Title] OR “ADHD” [Title] OR “attention deficit disorder” [Title] OR “hyperkin*” [Title] OR attention-deficit/hyperactivity disorder [Title]) AND (“child*” [Title] OR “young” [Title] OR “adolescent” [Title] OR “teenagers” [Title] OR “student*” [Title]) AND (“executive function” [Title] OR “executive functions” [Title] OR “inhibition” [Title] OR “interference” [Title] OR “cognitive control” [Title] OR “inhibitory control” [Title] OR “flexibility” [Title] OR “working memory” [Title] OR “switching” [Title] OR “shifting” [Title] OR “sustained attention” [Title] OR “attention” [Title])
and for Web of Science:
TI = (“exercise” OR “physical activity” OR “fitness” OR “physical exercise” OR “acute exercise” OR “chronic exercise” OR “aerobic” OR “resistance” OR “anaerobic” OR ”coordinative” OR “training”) AND TI = (“attention deficit hyperactivity disorder” OR “ADHD” OR “attention deficit disorder” OR “hyperkin*” OR attention-deficit/hyperactivity disorder) AND TI = (“child*” OR “young” OR “adolescent” OR “teenagers” OR “student*”) AND TI = (“executive function” OR “executive functions” OR “inhibition” OR “interference” OR “cognitive control” OR “inhibitory control” OR “flexibility” OR “working memory” OR “switching” OR “shifting” OR “sustained attention” OR “attention”).

We identified 449 studies from which we excluded 91 duplicates and further 347 that did not meet our inclusion criteria (<18 years old, diagnosed with ADHD, acute (one session) or long-term (>1 session) exercise intervention with control group or condition, assessing cognitive functions (either executive functions or attention) through automated or standardized neurocognitive test battery (i.e., not scales, clinician’s, teacher’s or parents reporting), peer-reviewed and available as full text in English, resulting in 11 studies identified. One inclusion and eight exclusions are in contrast with previous systematic reviews and accordingly warrant a brief explanation.

In contrast to a recent meta-analysis [70], we included data from Ziereis and Jansen [360] and Medina et al. [363]. The latter has previously been excluded from some [69,70] but not all [68,71] meta-analyses due to insufficient control conditions. We chose to include data from Medina et al. despite the possibility of test-retest effects caused by the non-randomized order of tests. The authors carried out task-familiarization prior to the first testing to accommodate this issue. Furthermore, we excluded data from studies published only as abstracts (e.g., [396]). We found the study from Ziereis and Jansen to comply with all our inclusion criteria. Also, in contrast to recent systematic reviews [68,367], we excluded three studies based on outcome variables. Craft was excluded since only measures of memory were included [85] and Piepmeier et al., and Pontifex et al., were both excluded based on the reported dependent variables. The outcome measures (‘total time to complete’ on the Stroop test, ‘Post-error slowing’ and ‘response accuracy’ across congruent and incongruent trials on a modified version of the Eriksen flanker Task) cannot be specified to any of our predefined categories [78,79]. This notwithstanding, the authors reported significant improvements across all three measures supporting the view that acute exercise improves cognitive functions in general. Furthermore, and also in contrast to a recent systematic analysis [367], we excluded one study that included not only children with ADHD, but also children diagnosed with disruptive behavior disorders (i.e., oppositional defiant disorder and conduct disorder) without an ADHD diagnosis [397].

Further seven studies were identified from references during reading resulting in a total of eighteen studies divided between investigations of effects of on cognitive functions after acute (*n* = 8) [74,75,76,77,81,82,83,363] and long-term (*n* = 10) [49,109,110,111,112,114,115,357,360,398] exercise. The identification of studies and inclusion process is presented in Figure A1.

We extracted ES as Hedges *g* to accommodate small sample sizes. Where these were not reported in tables or text but depicted in figures [49,78,115,360], we used a high-fidelity analytic tool (WebPlotDigitizer, V.4.1. Automeris, Austin, Texas, USA, 2018) to extract mean and standard deviations of post-tests. We did not pursue ESs when means or SDs were not reported or illustrated (e.g., reaction times in [115] and accuracy in [77]). Hedges’ g were computed for post measurements due to missing baseline data points for some studies [399]. Further, this approach was adopted to minimize the risk of inflating effect sizes due to catch-up effects for exercise groups with insignificantly lower baseline performances (see [356,400] for a discussion). We classified outcome measures as reflecting performance in one of following domains of executive control: (1) inhibitory control, (2) cognitive flexibility, (3) working memory as well as (4) sustained attention and (5) psychomotor speed (see Figure 2, Figure 3 and Figure 4). Importantly, we limited ES extracting to parameters that is readily allocated to one of the cognitive domains. As an example, we included two of three measures for cognitive functions from the recent RCT conducted by Benzing and Schmidt [398]. Here we extracted and plotted ES for reactions times in switch trials during the *Flanker task* as a measure for cognitive flexibility and total amount of correct answers in the *Color Span Backwards Test* assessing working memory. In contrast, we excluded the mean reaction time for corrects responses across congruent and incongruent trial for the *Simon task* as a measure of inhibition, since averaging across the congruent and incongruent conditions precludes assessment of changes specifically in inhibitory/interference control independent of processing or psychomotor speed. As discussed in Section 2.1 the cognitive domains are influenced from multiple, overlapping and interacting neuropsychological processes, which put both categorization and the construct validity of the associated neurocognitive test batteries into question. In case of parallel reports of raw data and results derived from the same data (e.g., reaction times for inconsistent trials vs the Stroop interference score), we plotted the test score best representing the domain (Stroop Interference score in this case). Furthermore, when both measures of RT and accuracy were reported, we extracted and plotted the former. As such, we cannot finitely reject the possibility that the some of the illustrated results reflect a change in strategy (i.e., a speed accuracy trade-off for PS) rather than a behavioral improvement per se. Supporting this decision, several of the included studies do not report both. If several independent measures of exercise-mediated changes within the same cognitive domain were reported, they are all included (e.g., Interference score from the Stroop test and No-Go true number from the go/no-go test [112]). In Table A1 and Table A2, we list included and excluded dependent variables from the studies presented in Figure 2, Figure 3 and Figure 4. Negative ESs signifying improved performance (e.g., decreased reaction times or number of errors) were reverse-coded. Consequently, all positive EFs depicted in Figure 2, Figure 3 and Figure 4 reflect improvements in performance.

**Table A1:** Included and excluded dependent variables from acute studies.

Study [#ref]	Type of Task	Included Measures	Cognitive Domain	Not Included	Extraction Method
Benzing et al., 2018 [77]	Modified Flanker Task, Modified Colour Span Backwards Task	Congruent RT, Inhibitory (incongruent) RT, Global Switch RT cost, Correct responses	PS, INH, CF, WM	Congruent accuracy, incongruent accuracy, switch accuracy, Global switch accuracy cost	Mean ± SD reported
Chang et al., 2012 [81]	Stroop, Wisconsin Card Sorting Task	Colour-word, perseverative errors	INH, CF	Word, Colour, total correct, perseverative responses, Non-Perseverative errors, Conceptual level, responses, categories completed,	Mean ± SD reported
Chuang et al., 2015 [75]	Go/No-Go	RT, Commission Error rate	PS, INH	Hit rate, Omission Error rate	Mean ± SD reported
Gawrilow et al., 2016 [74]	Modified Go/No-Go	Successful No-Go	INH	Overall errors on go-trials	Mean ± SD reported
Hung et al., 2016 [82]	Task-Switching Paradigm	Global Switch RT pure, Global Switch RT cost, Local Switch RT cost	PS, WM, CF	Global switch RT Mixed, Local Non Switch RT, Local Switch RT, Global Switch Accuracy Mixed, Local Switch RT Global Switch accuracy Pure, Local Non- Switch accuracy, local switch Accuracy, Global Switch Accuracy Cost, Local Switch Accuracy cost	Mean ± SD reported
Ludyga et al., 2018 [83]	Alternate Use	Fluency, Flexibility	CF, CF	Originality, Elaboration	Mean ± SD reported
Ludyga et al., 2017 [76]	Modified Flanker Task	RT congruent, RT incongruent	PS, INH	NA	Mean ± SD reported
Medina et al., 2010 [363]	Connor’s Continuous Performance Test II	Commission errors, Hit RT, Hit RT block Change	INH, PS, SA	Omission errors, Hit RT Standard Error, Variability, Detectability, Response style, Hit RT ISI change, Hit Standard Error interstimulus change, Perseverations, Hit standard error Block	Mean ± SD reported

Abbreviations: CF (Cognitive Flexibility), INH (Inhibitory Control), PS (Psychomotor Speed), RT (Reaction Time), SA (Sustained Attention), WM (Working Memory).

**Table A2:** Included and excluded dependent variables from chronic studies.

Study [#ref]	Type of Task	Included Measures	Cognitive Domain	Not Included	Extraction Method
Benzing & Schmidt 2019 [398]	Simon Task, modified Flanker Task, Colour Span Backwards task	Switch trials RT, correct Responses	CF, WM	RT across trials	Mean ± SD reported
Chang et al., 2014 [115]	Go/No-Go	Accuracy no-go trials	INH	Accuracy go trials	Mean ± SD from figures.
Choi et al., 2015 [114]	Wisconsin Card Sorting Test	Perseverative Errors	CF	NA	Mean ± SD reported
Kang et al., 2011 [49]	Trail Making Test part b	Time to completion	CF	Digit Symbol Task	Mean ± SD from figures
Lee et al., 2017 [357]	Stroop	Interference	INH	Colour-Word	Mean ± SD reported
Memarmoghaddam et al., 2016 [112]	Stroop, Go/No-Go	Consistent RT, Interference, No-Go true number, True RT	PS, INH, INH, PS	Consistent and inconcistent error number, consistent and inconcistent no reponses, consistent and inconcistent true number, inconsistent RT, Go and no-go true number, Go and no-go error number, error RT	Mean ± SD reported
Pan et al., 2015 [111]	Stroop, Wisconsin Card Sorting Test	Colour-word, Perseverative errors	INH, CF	Total correct, perseverative responses, non-perseverative errors, conceptual levels, Responses, categories completed	Mean ± SD reported
Pan et al., 2016 [110]	Stroop	Colour word	INH	NA	Mean ± SD reported
Verret et al., 2012 [109]	Sky Search	Score pondering, walk/don’t walk pondering	SA, INH	Time targeted pondering, attention pondering, sky search DT pondering	Mean ± SD reported
Ziereis & Jansen 2015 [360]	Digit Span, Letter-Number Sequencing	Digit span Index score, Letter-number sequencing index score	WM, WM	Backwards digit span, forward digit span	Mean ± SD from figures

Abbreviations: CF (Cognitive Flexibility), INH (Inhibitory Control), PS (Psychomotor Speed), RT (Reaction Time), SA (Sustained Attention), WM (Working Memory).
